# Health of Public Reformatories

**Published:** 1875-02-01

**Authors:** 


HEALTH OF PUBLIC REFORMATORIES.—At a recent meeting of the State Board of Health of Michigan, Dr. R. E. Kedzie reported the results of his examination of the different reformatory institutions of that state, with special reference to their ventilation and water supply. It is stated, that in all of them, he found the ventilation bad, the air yielding from fourteen to thirty-two parts of carbonic acid gas in ten thousand parts of air, and the water more or less foul. It is to be hoped that his recommendations of legislative action will be speedily complied with by the lawmakers of that state.

